# Clinical Evaluation of Ozone Gel Versus Hyaluronic Acid Gel on Palatal Wound Following Free Gingival Graft Harvesting: A Randomized Clinical Trial

**DOI:** 10.1155/ijod/4177557

**Published:** 2025-12-12

**Authors:** Hisham Tarek, Nesma Shemais, Dalia Ghalwash, Ahmed El Barbary

**Affiliations:** ^1^ Oral Medicine and Periodontology Department, Faculty of Dentistry, Cairo University, Cairo, Egypt, cu.edu.eg; ^2^ Oral Medicine and Periodontology Department, Faculty of Dentistry, The British University in Egypt, El Sherouk, Egypt, bue.edu.eg; ^3^ Department of Preventive Dental Sciences, College of Dentistry, Gulf Medical University, Ajman, UAE, gmu.ac.ae; ^4^ Oral Medicine and Periodontology, Faculty of Dentistry, Galala university, Suez, Egypt, gu.edu.eg

**Keywords:** free gingival graft, hyaluronic acid, ozone therapy, pain management, palatal wound healing, periodontal surgery, wound healing

## Abstract

**Objective:**

This randomized clinical trial (RCT) compares the effectiveness of ozone gel (GeliO3) and hyaluronic acid (HA) gel (Gengigel) in enhancing wound healing and reducing postoperative discomfort following free gingival graft (FGG) harvesting.

**Methods:**

Fifty‐six patients requiring FGG for mucogingival defects were randomly assigned into two groups: the ozone gel group and the HA gel group. The primary outcome was postoperative pain, assessed using the visual analog scale (VAS) and analgesic consumption. Secondary outcomes included wound healing, evaluated using the Landry healing index, and color match assessment. Data were analyzed using appropriate statistical tests with a significance level of *p* < 0.05.

**Results:**

Both treatment groups exhibited significant pain reduction over time (*p* < 0.001). Although there was no statistically significant difference between the groups, a faster decline in pain was observed in the ozone group by Day 3. Analgesic consumption was significantly lower in the ozone group on Days 2 and 3 (*p* = 0.042). The healing index and color match scores showed a steady improvement in both groups, with the ozone group demonstrating slightly higher values at various time points, though not statistically significant.

**Conclusion:**

Both ozone gel and HA gel effectively enhanced post‐FGG healing, reduced pain, and improved tissue esthetics. Ozone therapy showed potential advantages in early pain relief and lower analgesic dependence. These findings suggest that ozone therapy may serve as an alternative or adjunctive treatment for palatal wound management in periodontal surgery. Further research is needed to confirm its clinical superiority over HA gel.

## 1. Introduction

Free gingival grafting is a widely utilized procedure in periodontal therapy aimed at improving soft tissue stability, increasing the band of keratinized gingiva, and enhancing periodontal health [1]. Initially introduced in the 1960s, this technique continues to be a preferred approach for gingival augmentation in both natural dentition and implant cases [2]. Despite its effectiveness, one of the primary challenges associated with FGG is donor site morbidity, including extended healing periods, postoperative pain, and a higher risk of infection due to secondary intention healing [3].

The healing of the palatal donor site progresses through four distinct stages: hemostasis, inflammation, proliferation, and maturation [1]. Although oral mucosa typically exhibits faster healing than skin due to its unique biological properties, the donor site of an FGG is particularly prone to delayed re‐epithelialization and prolonged inflammation [4]. Several methods, including hemostatic agents, platelet‐rich fibrin (PRF), and bioactive gels, have been proposed to accelerate wound healing and alleviate postoperative pain [5]. Among these, hyaluronic acid (HA) gel and ozone therapy have emerged as promising adjuncts due to their regenerative potential and anti‐inflammatory characteristics [6, 7].

HA is a naturally occurring polysaccharide that plays a critical role in tissue hydration, cell proliferation, and extracellular matrix remodeling. Due to its viscoelastic properties, HA forms a protective barrier over wound sites, preventing microbial contamination while expediting epithelialization [6]. Clinical studies have demonstrated that HA application significantly reduces postsurgical pain, improves color match at the donor site, and minimizes inflammation compared to standard healing methods [6, 8]. Furthermore, HA’s regulatory effects on cytokine activity and its ability to promote fibroblast proliferation suggest that it may contribute to long‐term wound stability, making it a valuable component in regenerative periodontal therapies [9].

Meanwhile, ozone therapy has been recognized for its antimicrobial, anti‐inflammatory, and oxygenating properties, which enhance wound healing [7]. Ozone, a triatomic oxygen molecule (O_3_), induces oxidative stress that stimulates cellular metabolism, enhances blood vessel formation, and reduces microbial load at the wound site. Clinical research has shown that ozone therapy can significantly decrease pain perception and promote faster wound closure when compared to conventional periodontal dressings [10, 11]. Additionally, ozone supports increased oxygenation of tissues, which is essential for the initial stages of healing, particularly in palatal donor sites where vascular supply plays a crucial role in tissue regeneration [11]. Although both HA and ozone therapy have been individually studied for their beneficial effects on wound healing, direct comparative clinical evidence evaluating their efficacy in palatal wound healing post‐FGG harvesting remains limited. The current research aims to close the gap by conducting a randomized controlled clinical trial to assess the effectiveness of ozone gel compared to HA gel in promoting the healing of palatal donor sites. Key clinical parameters, including postoperative pain (measured as the primary outcome), wound healing rate, color match, and patient‐reported comfort (measured as secondary outcomes), were thoroughly analyzed.

## 2. Material and Methods

**Figure 1:**
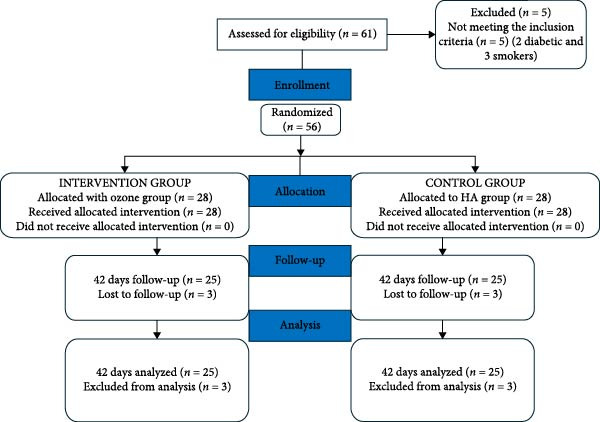
Consort diagram.

The present randomized clinical trial (RCT) enrolled 56 patients, age ranged between 18 and 65 years, with mucogingival defects in need for free gingival graft (FGG). Patients randomly were assigned into group of two groups: the control group receiving the HA gel (Gengi gel) and the intervention group that received Ozone gel (GeliO3). Participants were selected from the outpatient clinic of the Department of Oral Medicine and Periodontology at the Faculty of Dentistry, Cairo University, between December 2022 and February 2025. The trial protocol was registered on https://www.clinicaltrials.gov protocol registration on December 26, 2022 (with an identifier ID: NCT05690100). The research protocol was approved by the Ethics Committee of the Faculty of Dentistry, Cairo University, in November 2022 (Approval Number:11‐10‐22). Informed consents were then obtained from all participants prior to their inclusion in the study. The trial was conducted in accordance with the principles of the Helsinki Declaration of 1975, as updated in 2013, and was reported following the CONSORT guidelines [12]. The patient inclusion, allocation, follow‐up, and analysis process is summarized in the CONSORT flow diagram (Figure 1).

### 2.1. Study Population

Exclusion criteria encompassed individuals who smoked, had diabetes mellitus, were pregnant or lactating, or suffered from systemic diseases such as hypertension, autoimmune disorders, hepatic or renal impairments, and cardiovascular conditions. Patients receiving medications known to alter wound healing, including corticosteroids, anticoagulants, and immunosuppressants, were also excluded. Only systemically healthy, nonsmoking individuals were included to eliminate potential confounding variables and ensure uniform healing conditions.

### 2.2. Sample Size, Randomization, and Blinding

The sample size was determined based on data from a previous study [6], assuming a normal distribution of responses within each subject group with a standard deviation (SD) of 2.27 mm. The alpha level was set at 0.05, and the power at 0.8, with an effect size (d) of 1.5 for Student’s *t*‐test. As a result, 25 patients were required per group. To account for a projected 10% dropout rate after 42 days, the sample size was increased to 28 subjects per group, totaling 56 participants. The calculation was performed using G‐Power software Version 3.1.2 (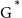
Power: statistical power analysis program, Version 3.1., Heinrich‐Heine‐Universität, Düsseldorf, Germany).

Sequence generation was executed using simple randomization by a random allocation software by an investigator (AB) who was not involved in neither recruitment nor treatment procedures. Allocation concealment was conducted by the same investigator (AB) using sequentially‐numbered, opaque, sealed envelopes handled to the surgeon (HT) who did not open them until the beginning of the surgical procedure. Participants were then assigned to either the Ozone gel (GeliO3) (test group) or HA gel (Gengigel) (control group). The outcome assessors (NS) and the biostatistician remained blinded, while the principal investigator and the patients were informed of the treatment only on the day of surgery.

### 2.3. Treatment Protocol

Presurgical Phase; fullmouth professional mechanical plaque removal (PMPR) was performed using ultrasonic scalers (Woodpecker UDS‐P with LED, China) and hand instruments such as Gracey curettes, After Five Gracey curettes, and universal curettes (Hu‐Friedy, Chicago, IL, USA). Additionally, rubber polishing pads and prophylaxis paste (Alpha‐Pro Prophylaxis Paste, USA) were used for polishing. The root surfaces were carefully debrided to ensure a smooth and firm consistency. Patients were provided with proper oral hygiene instructions, which included brushing twice daily with a soft toothbrush. Chemical plaque control was also recommended, with the prescription of a 0.125% Chlorhexidine HCL mouthwash to be used twice daily for 2 weeks.

#### 2.3.1. Preparation of the Recipient Site

For both study groups, the recipient site preparation followed the Camargo et al. technique [13], the site was first assessed, and the required augmentation area was outlined using a periodontal probe or surgical marker. A horizontal incision was made 1 mm coronal to the cementoenamel junction (CEJ) of adjacent teeth, extending at least 3 mm mesiodistally beyond the defect. Two vertical incisions were then created in an apicocoronal direction, extending 4–5 mm beyond the mucogingival junction. A split‐thickness dissection was performed using a scalpel or microsurgical instruments, carefully separating the epithelium and connective tissue while preserving the underlying periosteum. The epithelial layer of the alveolar mucosa was excised, exposing a vascularized connective tissue bed necessary for graft revascularization. Any granulation tissue, muscle fibers, or excessive fibrotic tissue was meticulously removed with curettes or microsurgical scissors to optimize healing.

### 2.4. Harvesting the FGG

In both groups, FGG harvesting from the palate followed the technique described by Zucchelli et al. [14]. The procedure began with two horizontal incisions, where the coronal incision was made 2 mm apical to the gingival margin of the adjacent teeth, while the two vertical incisions were created to define the graft area. A 15c blade was inserted along the coronal horizontal incision at one edge, perpendicular to the bone. Once the desired graft thickness of 1–1.5 mm was achieved, the blade’s direction was adjusted to be parallel to the hard palate and moved mesiodistally to elevate the graft on one side. This process continued until the graft was completely detached from the palate, ensuring uniform thickness while moving apically with the blade. Care was taken to preserve the palatal periosteum.

After harvesting, the graft was immediately placed on sterile gauze moistened with saline. Afterwards, the FGG was positioned and securely adapted to the recipient site, stabilized using two simple interrupted periosteal sutures and a criss‐cross sling suture with 5–0 Vicryl suture material (M‐Nature Sutures; International Sutures Manufacturing Co., Egypt). The suturing was performed using a Castroviejo needle holder (Nordent Manufacturing Inc., USA) to ensure proper fixation.

### 2.5. Management of the Palatal Wound

Following harvesting the FGG, bleeding was controlled, when present, by applying pressure with sterile gauze for 5 min until it stopped.

#### 2.5.1. Group I (Ozone‐Test Group)

In the intervention group, GeliO3 (Bio‐ozonized olive oil, 20 mEq O_2_/kg; Bioemmei Srl, Vicenza, Italy) was applied ~0.3–0.5 mL of ozonated olive oil gel (GeliO3, Bioemmei Srl, Italy; 20 mEq O_2_/kg) was applied uniformly to the palatal wound using a 3 mL sterile syringe disposable plastic syringe and a blunt 23‐gauge needle immediately after graft harvesting. The palatal wound was then immediately covered with a noneugenol periodontal pack (Pericem, Pericem cement Quengco, Italy) three days postoperatively, patients were recalled for evaluation, during which the periodontal pack was removed to assess the healing of the palatal wound. Ozone was then reapplied, and the donor site was once again covered with the periodontal pack, as shown in Figure 2.

Figure 2Clinical photographs for the ozone group healing: (a) Day 0, (b) Day 3, (c) Day 7, (d) Day 14, (e) Day 21, and (f) Day 42.

**Figure:**
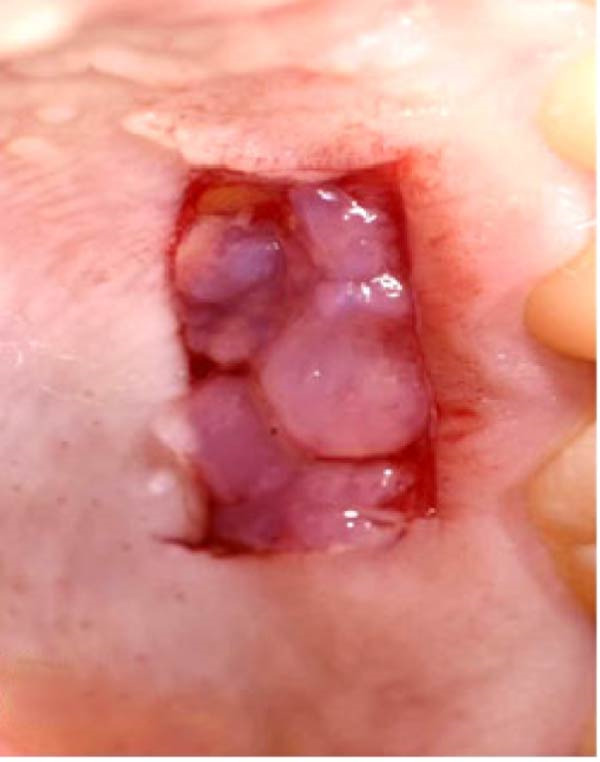
(a)

**Figure:**
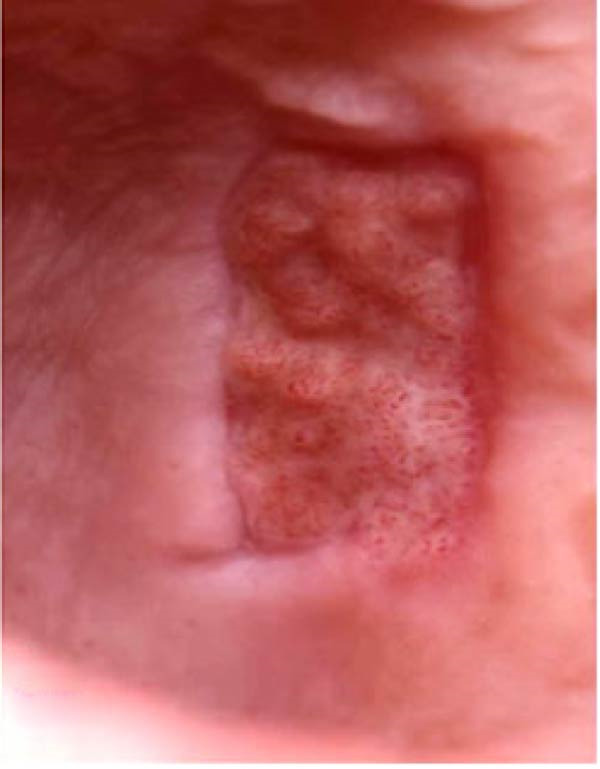
(b)

**Figure:**
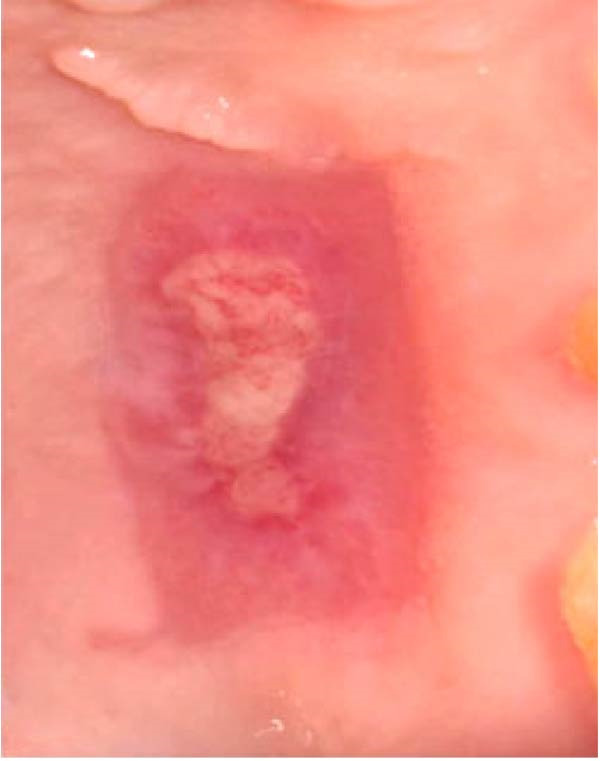
(c)

**Figure:**
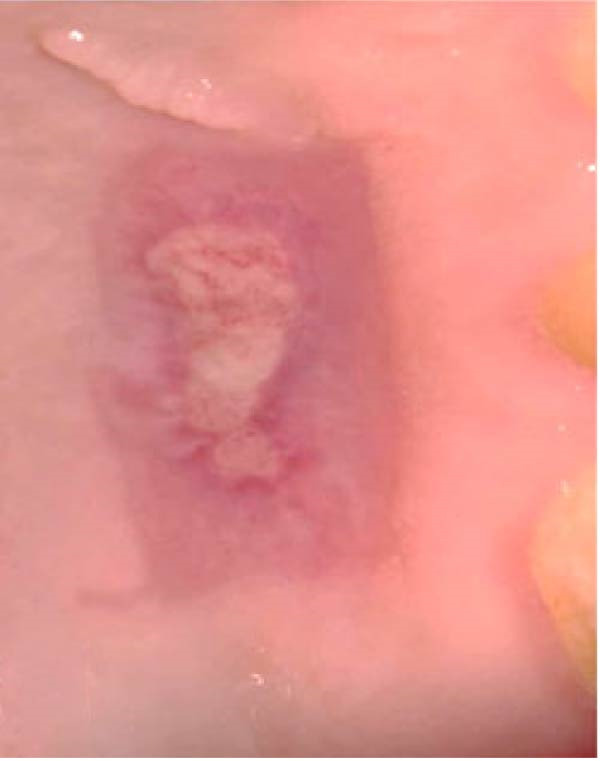
(d)

**Figure:**
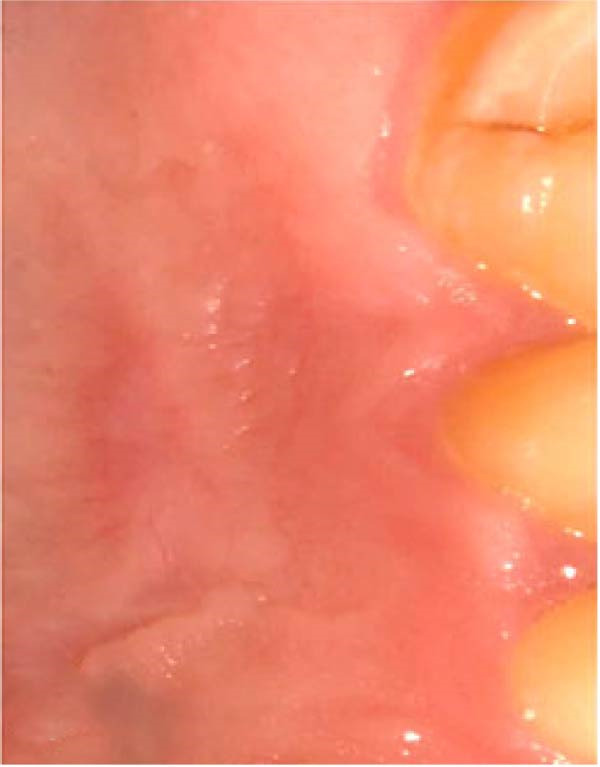
(e)

**Figure:**
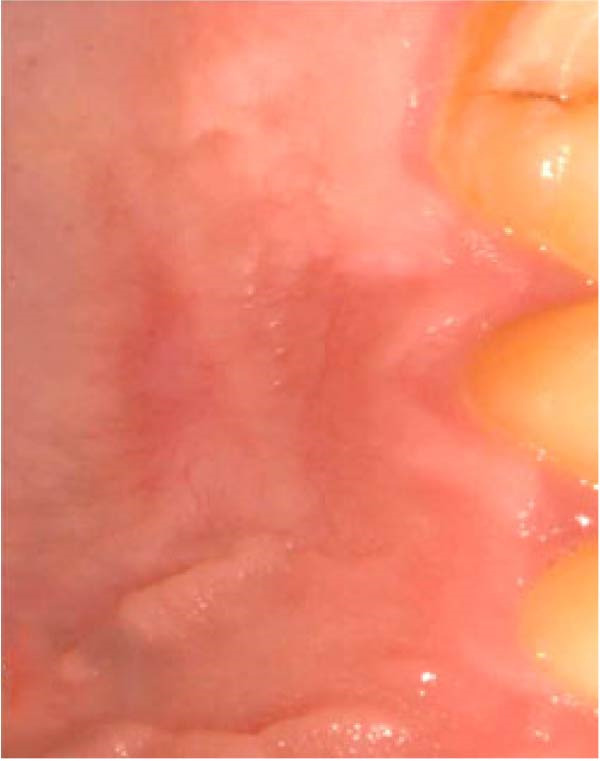
(f)

#### 2.5.2. Group II (0.2% HA–Control Group)

In the control group, 0.2% HA gel (Gengigel, Ricerfarma S.r.l., Milano, Italy) was applied ~0.3–0.5 mL uniformly to the palatal wound using a 3 mL sterile syringe disposable plastic syringe and a blunt 23‐gauge needle immediately after graft harvesting. The palatal wound was immediately covered with a noneugenol periodontal pack (Pericem). 3 days postoperatively, patients were recalled for wound evaluation, and the periodontal pack was removed. The 0.2% HA gel was then reapplied, and the donor site was repacked with the periodontal pack, as shown in Figure 3.

Figure 3Clinical photographs for the HA group: (a) Day 0, (b) Day 3, (c) Day 7, (d) Day 14, (e) Day 21, and (f) Day 42.

**Figure:**
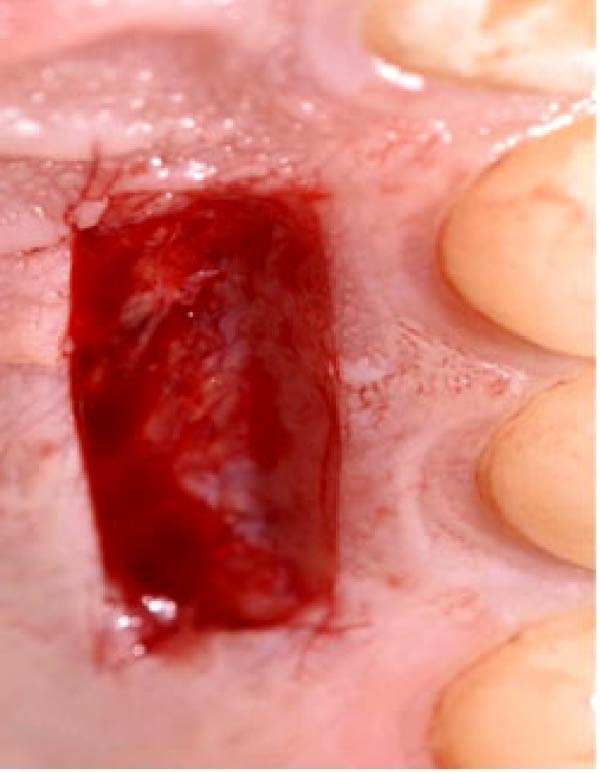
(a)

**Figure:**
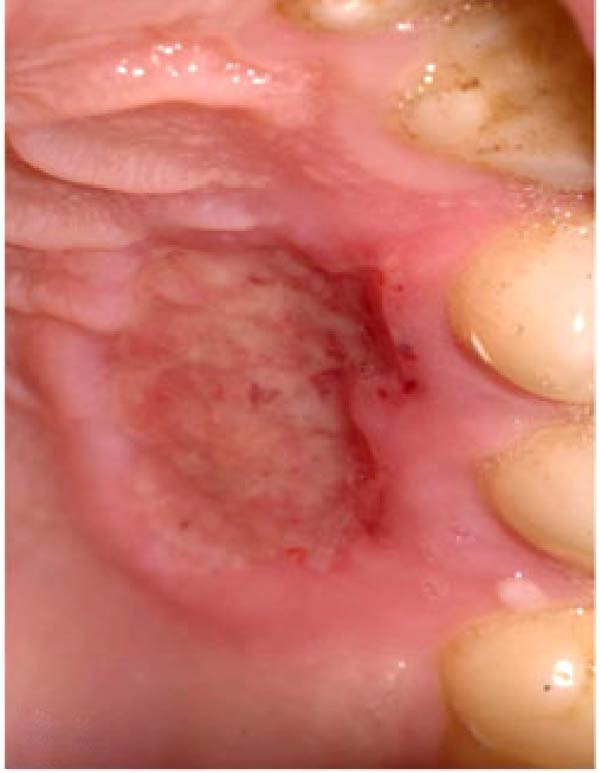
(b)

**Figure:**
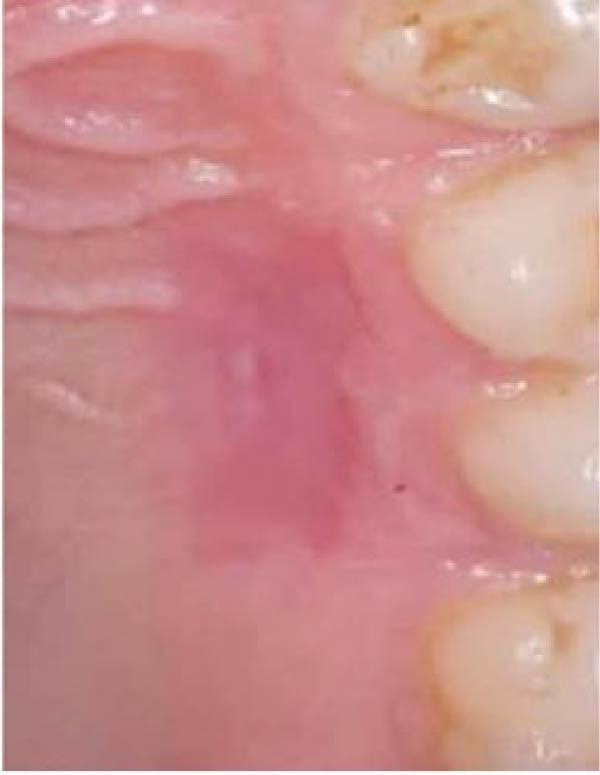
(c)

**Figure:**
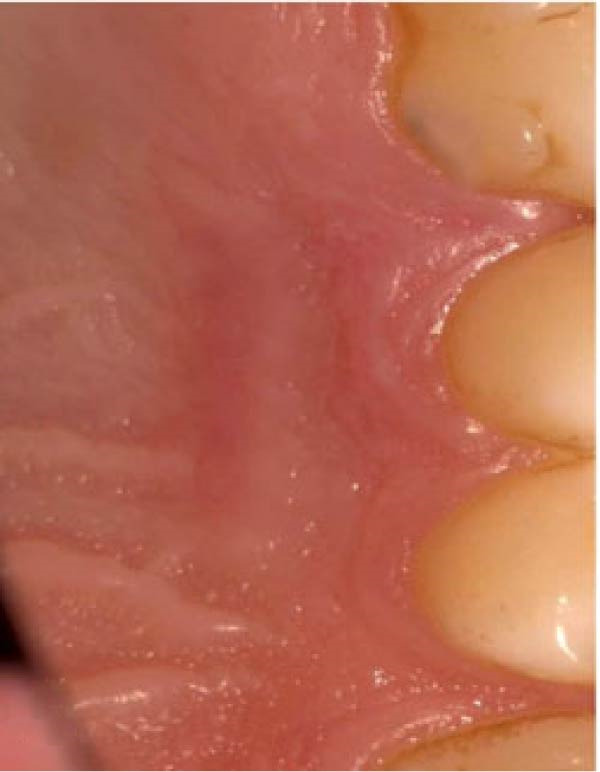
(d)

**Figure:**
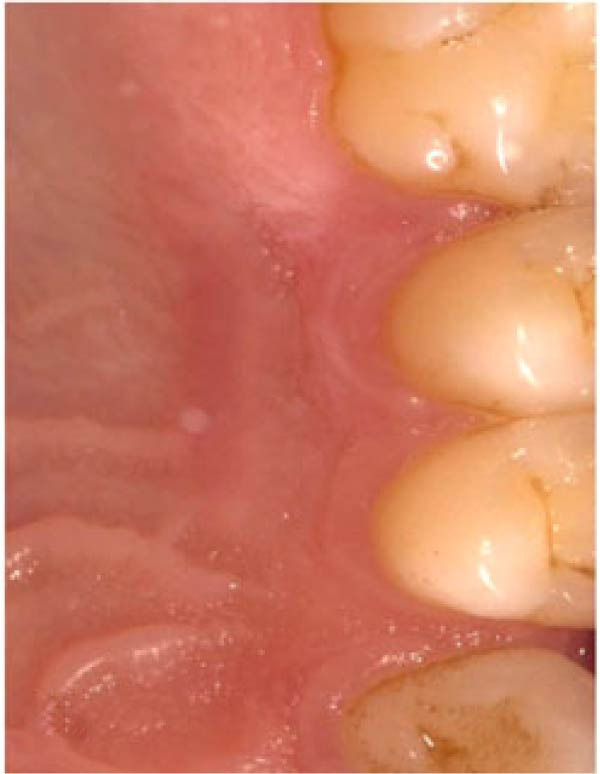
(e)

**Figure:**
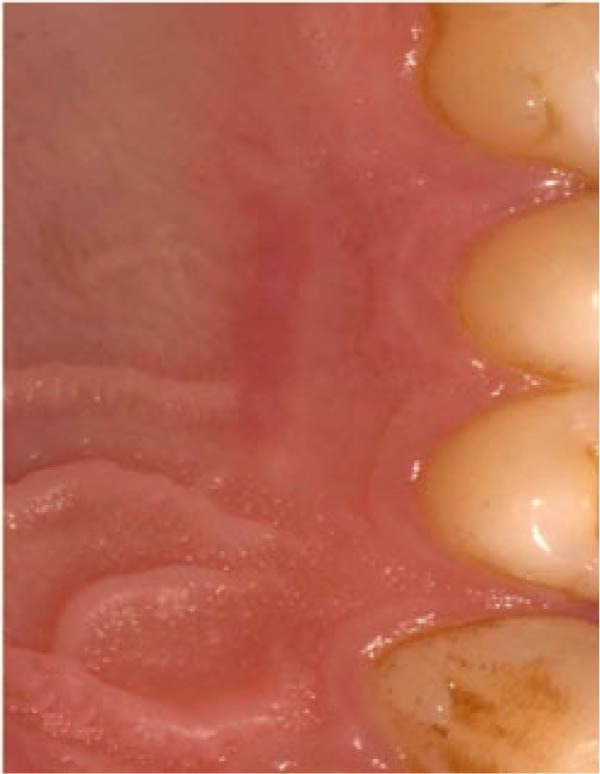
(f)

### 2.6. Clinical Outcomes

The primary outcomes of this study included postoperative pain assessment through the visual analog scale (VAS) and mean analgesic consumption [15]. Patients reported their pain levels directly using the VAS, which ranged from 0 to 10 (0 indicating no pain, 1 minimal pain, 5 moderate pain, and 10 severe pain) [15]. Pain scores were recorded daily for 1 week to evaluate postoperative discomfort [15]. Additionally, postoperative pain was indirectly assessed by measuring the mean consumption of analgesics over a period of 7 days, recorded in milligrams [16].

The secondary outcomes focused on soft tissue healing and color match of the palatal mucosa. The healing of the donor site was evaluated using Landry et al.’s Hhealing Iindex [17] which assesses tissue recovery based on mucosal color, bleeding upon palpation, presence of granulation tissue, epithelialization of the incision margin, and suppuration. Healing was scored on a 5‐point scale, where 1 indicated very poor healing and 5 represented excellent healing. Additionally, the color match of the palatal mucosa was assessed on Days 3, 7, 14, 21, and 42 by comparing it to the adjacent and opposite mucosa. This evaluation utilized an objective VAS (0–10 scale), where 0 represented no color match and 10 indicated an excellent color match with the surrounding tissues. The assessments were conducted by the main supervisor, who was blinded to the treatment group assignment, ensuring an objective evaluation of the healing process.

### 2.7. Postoperative Care and Follow‐Up Visits

The periodontal pack was removed after 1 week. On the day of surgery, patients were administered 600 mg of Ibuprofen (Abbott, Egypt) for pain management and were instructed to take an additional 600 mg dose only as needed, recording the number of pills consumed to indirectly assess postoperative pain through mean analgesic consumption (mg). To promote oral hygiene and reduce the risk of infection, patients were advised to rinse with an antiseptic mouth rinse (0.12% Chlorhexidine HCL) twice daily for 1 min for 2 weeks following surgery. Additionally, they were instructed to avoid vigorous brushing and trauma to the surgical site for 3 weeks. After this period, patients were advised to gently brush the treated area using a soft toothbrush and a circular scrubbing technique.

Sutures were removed 14 days postoperatively from the recipient site where the FGG was placed. To monitor the healing progress of the palatal wound, clinical photographs were taken at multiple time points: on Day 3, then at 1, 2, and 3 weeks, and finally on Day 42 postoperatively. These images were used to evaluate the healing process over time.

### 2.8. Photographic Evaluation Protocol

Clinical photographs were captured using a standardized digital photographic protocol to ensure reproducibility and reduce bias. A digital single‐lens reflex (DSLR) camera (Canon EOS 700D, Canon Inc., Tokyo, Japan), equipped with an EF‐S 60 mm macro lens, was securely mounted on a tripod to maintain consistency. The photographic settings (ISO 200, aperture f/22, shutter speed 1/125 s) remained constant throughout the study. All photographs were acquired from a standardized distance of ~30 cm, with the camera positioned perpendicularly to the palatal plane. Uniform lighting conditions were ensured by employing a ring LED flash with consistent intensity across all images [18, 19]. Prior to initiating the study, the examiner responsible for photographic assessment underwent rigorous calibration sessions involving standardized image assessments and periodic recalibration throughout the study to maintain consistency in image evaluation.

### 2.9. Statistical Analysis

Numerical data were presented as mean, SD, median, minimum, maximum, and interquartile range (IQR) values. They were explored for normality by checking the data distribution and using Shapiro–Wilk’s test. Age data were normally distributed and were analyzed using an independent *t*‐test. Other data were nonparametric. They were analyzed for intergroup comparisons using the Mann–Whitney *U* test and for intragroup comparisons using Freidman’s and Nemenyi’s tests. *p*‐values were adjusted for multiple comparisons using the False Discovery Rate (FDR) method. Correlations were analyzed using Spearman’s rank‐order correlation coefficient. The significance level was set at *p* < 0.05. statistical analysis was performed with R statistical analysis software version 4.4.2 for Windows.1 1R.

## 3. Results

**Figure 4:**
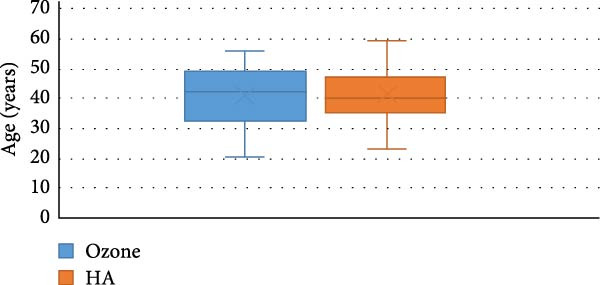
Stacked bar chart for gender distribution.

**Figure 5:**
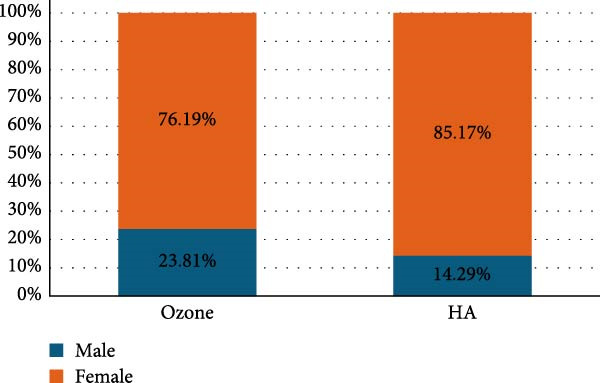
Box plot for age values.

The baseline characteristics of the 50 participants, divided into two groups, are presented in Table 1. Intergroup comparisons and summary statistics for demographic data are presented in Table 1 and Figures 4 and 5.

**Table 1:** Intergroup comparisons and summary statistics for demographic data.

Parameter	Measurement	Ozone	HA	*p*‐Value
Gender (*n* [%])	Male	7 (23.81%)	5 (14.29%)	0.697 ns
	Female	18 (76.19%)	20 (85.71%)	
Age (years)	Mean ± SD	40.67 ± 9.90	41.14 ± 9.34	0.873 ns
	Median (IQR)	42.00 (15.00)	40.00 (9.00)	

Abbreviation: ns, not significant.

**Figure 6:**
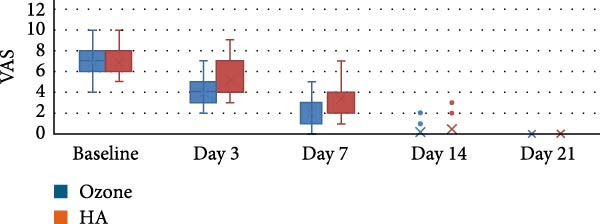
Box plot for VAS values.

**Figure 7:**
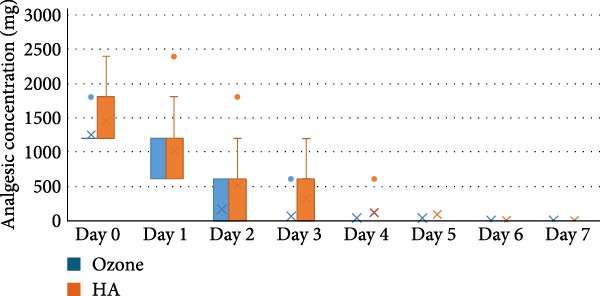
Box plot for analgesic concentration (mg) values.

**Figure 8:**
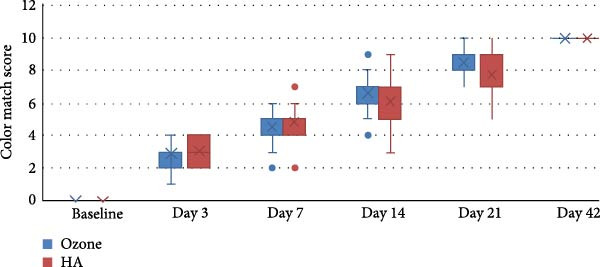
Box plot for color match score values.

**Figure 9:**
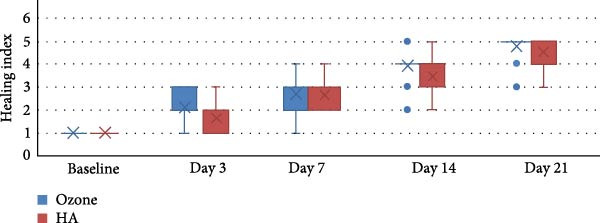
Box plot for healing index values.

The study comprised 50 cases that were randomly and equally allocated to each of the studied groups. There were seven males and 18 females with a mean age of (40.67 ± 9.90) years in the ozone group. In contrast, there were five males and 20 females in the HA group with a mean age of (41.14 ± 9.34) years. There was no significant difference between both groups regarding gender (*p* = 0.697) and age (*p* = 0.873). VAS are presented in Table 2 with intergroup comparisons in Table 3. Within different intervals, there was no statistically significant difference in pain scores measured in both groups (*p* > 0.05). For Day 7, the effect size was moderate, however, for other intervals, the effect size was small. At baseline, the ozone group (7) had a higher median than the HA group (6). In contrast, on Day 3, the HA group (5) had a higher median than the ozone group (4). Starting from Day 14, the medians of both groups were zero. Descriptive statistics for analgesic concentration (mg) are presented in Table 4. Intergroup comparisons and summary statistics for analgesic concentration (mg) are presented in Table 5 and Figure 6. On Day 2, the median analgesic concentration in the HA group (600 mg) was significantly higher than that of the ozone group (0) mg (*p* = 0.042), with a moderate effect size. On Day 3, although both groups had the same median value (0) mg, the difference remained statistically significant (*p* = 0.042) with a moderate effect due to the large variability between groups. The ozone group had an IQR of (0), indicating minimal dispersion, whereas the HA group had an IQR of (600) mg, indicating a wide distribution of values. For other intervals, both groups had the same median values, the differences were not statistically significant (*p* > 0.05), and the effect sizes were small. Palatal healing index is presented in descriptive form in Table 6. Intergroup comparisons and summary statistics for the healing index are presented in Table 7 and Figure 7. Within different intervals, there was no statistically significant difference in healing index values measured in both groups (*p* > 0.05). For Days 3 and 14, the effect size was moderate; however, for other intervals, the effect sizes were small. At baseline (1), on Day 3 (2), Day 7 (3), and Day 21 (5), both groups had the same median. On Day 14, the ozone group (4) had a higher median than the HA group (3). Descriptive statistics for color match scores are presented in Table 8 and the overall distribution of color match values is depicted in the box‐plot chart (Figure 8). Intergroup comparisons and summary statistics for color match score are presented in Table 9 and Figure 9. Within different intervals, there was no statistically significant difference in color scores measured in both groups (*p* > 0.05), with the effect size being small. At baseline, both groups had a median of zero. On Day 3, both had a median score of (3). Similarly, on Day 7, both had a score of (5). On Days 14 and 21, the ozone group (7 and 9) had higher medians than the HA group (6 and 8), respectively. Finally, on Day 42, both groups had a median of 10. (1), on Day 3 (2), Day 7 (3), and Day 21 (5), both groups had the same median. On Day 14, the ozone group (4) had a higher median than the HA group (3).

**Table 2:** Descriptive statistics for VAS.

Group	Time	Mean	SD	Median	Minimum	Maximum
Ozone	Baseline	6.90	1.84	7.00	4.00	10.00
	Day 3	4.48	1.60	4.00	2.00	7.00
	Day 7	2.14	1.35	2.00	0.00	5.00
	Day 14	0.24	0.54	0.00	0.00	2.00
	Day 21	0.00	0.00	0.00	0.00	0.00

HA	Baseline	6.90	1.61	6.00	5.00	10.00
	Day 3	5.24	1.81	5.00	3.00	9.00
	Day 7	3.33	1.88	3.00	1.00	7.00
	Day 14	0.52	0.98	0.00	0.00	3.00
	Day 21	0.00	0.00	0.00	0.00	0.00

**Table 3:** Intergroup comparisons and summary statistics for VAS.

Time	Measurement	VAS		*p*‐Value	Effect size	
		Ozone	HA		(95% CI)	Magnitude
Baseline	Mean ± SD	6.90 ± 1.84	6.90 ± 1.61	0.990 ns	0.004 (0.004:0.360)	Small
	Median (IQR)	7.00 (2.00)	6.00 (2.00)			
Day 3	Mean ± SD	4.48 ± 1.60	5.24 ± 1.81	0.410 ns	0.197 (0.010:0.460)	Small
	Median (IQR)	4.00 (2.00)	5.00 (3.00)			
Day 7	Mean ± SD	2.14 ± 1.35	3.33 ± 1.88	0.180 ns	0.312 (0.040:0.570)	Moderate
	Median (IQR)	2.00 (2.00)	3.00 (2.00)			
Day 14	Mean ± SD	0.24 ± 0.54	0.52 ± 0.98	0.703 ns	0.100 (0.007:0.370)	Small
	Median (IQR)	0.00 (0.00)	0.00 (0.00)			
Day 21	Mean ± SD	0.00 ± 0.00	0.00 ± 0.00	NA	NA	NA
	Median (IQR)	0.00 (0.00)	0.00 (0.00)			

Abbreviations: NA, not applicable; ns, not significant.

**Table 4:** Descriptive statistics for analgesic concentration (mg).

Group	Time	Mean	SD	Median	Minimum	Maximum
Ozone	Day 0	1257.14	180.48	1200.00	1200.00	1800.00
	Day 1	828.57	298.57	600.00	600.00	1200.00
	Day 2	171.43	277.75	0.00	0.00	600.00
	Day 3	57.14	180.48	0.00	0.00	600.00
	Day 4	28.57	130.93	0.00	0.00	600.00
	Day 5	28.57	130.93	0.00	0.00	600.00
	Day 6	0.00	0.00	0.00	0.00	0.00
	Day 7	0.00	0.00	0.00	0.00	0.00

HA	Day 0	1457.14	405.67	1200.00	1200.00	2400.00
	Day 1	1028.57	573.71	600.00	600.00	2400.00
	Day 2	514.29	475.69	600.00	0.00	1800.00
	Day 3	314.29	407.78	0.00	0.00	1200.00
	Day 4	114.29	241.42	0.00	0.00	600.00
	Day 5	85.71	215.14	0.00	0.00	600.00
	Day 6	0.00	0.00	0.00	0.00	0.00
	Day 7	0.00	0.00	0.00	0.00	0.00

**Table 5:** Intergroup comparisons and summary statistics for analgesic concentration (mg).

Time	Measurement	Analgesic conc. (mg)		*p*‐Value	Effect size	
		Ozone	HA		(95% CI)	Magnitude
Day 0	Mean ± SD	1257.14 ± 180.48	1457.14 ± 405.67	0.114 ns	0.296 (0.040:0.530)	Small
	Median (IQR)	1200.00 (0.00)	1200.00 (600.00)			
Day 1	Mean ± SD	828.57 ± 298.57	1028.57 ± 573.71	0.351 ns	0.146 (0.006:0.410)	Small
	Median (IQR)	600.00 (600.00)	600.00 (600.00)			
Day 2	Mean ± SD	171.43 ± 277.75	514.29 ± 475.69	0.042 ^∗^	0.406 (0.140:0.660)	Moderate
	Median (IQR)	0.00 (600.00)	600.00 (600.00)			
Day 3	Mean ± SD	57.14 ± 180.48	314.29 ± 407.78	0.042 ^∗^	0.382 (0.110:0.620)	Moderate
	Median (IQR)	0.00 (0.00)	0.00 (600.00)			
Day 4	Mean ± SD	28.57 ± 130.93	114.29 ± 241.42	0.248 ns	0.218 (0.020:0.450)	Small
	Median (IQR)	0.00 (0.00)	0.00 (0.00)			
Day 5	Mean ± SD	28.57 ± 130.93	85.71 ± 215.14	0.351 ns	0.160 (0.000:0.390)	Small
	Median (IQR)	0.00 (0.00)	0.00 (0.00)			
Day 6	Mean ± SD	0.00 ± 0.00	0.00 ± 0.00	NA	NA	NA
	Median (IQR)	0.00 (0.00)	0.00 (0.00)			
Day 7	Mean ± SD	0.00 ± 0.00	0.00 ± 0.00	NA	NA	NA
	Median (IQR)	0.00 (0.00)	0.00 (0.00)			

Abbreviations: NA, not applicable; ns, not significant.

^∗^Significant (*p*<0.05).

**Table 6:** Descriptive statistics for the healing index.

Group	Time	Mean	SD	Median	Minimum	Maximum
Ozone	Baseline	1.00	0.00	1.00	1.00	1.00
	Day 3	2.10	0.70	2.00	1.00	3.00
	Day 7	2.71	0.96	3.00	1.00	4.00
	Day 14	3.90	0.94	4.00	2.00	5.00
	Day 21	4.76	0.62	5.00	3.00	5.00

HA	Baseline	1.00	0.00	1.00	1.00	1.00
	Day 3	1.62	0.67	2.00	1.00	3.00
	Day 7	2.62	0.67	3.00	2.00	4.00
	Day 14	3.43	0.75	3.00	2.00	5.00
	Day 21	4.52	0.75	5.00	3.00	5.00

**Table 7:** Intergroup comparisons and summary statistics for healing index.

Time	Measurement	Healing index		*p*‐Value	Effect size	
		Ozone	HA		Eta2 (h) (95% CI)	Magnitude
Baseline	Mean ± SD	1.00 ± 0.00	1.00 ± 0.00	NA	NA	NA
	Median (IQR)	1.00 (0.00)	1.00 (0.00)			
Day 3	Mean ± SD	2.10 ± 0.70	1.62 ± 0.67	0.072 ns	0.333 (0.060:0.600)	Moderate
	Median (IQR)	2.00 (1.00)	2.00 (1.00)			
Day 7	Mean ± SD	2.71 ± 0.96	2.62 ± 0.67	0.517 ns	0.102 (0.006:0.390)	Small
	Median (IQR)	3.00 (1.00)	3.00 (1.00)			
Day 14	Mean ± SD	3.90 ± 0.94	3.43 ± 0.75	0.072 ns	0.325 (0.040:0.590)	Moderate
	Median (IQR)	4.00 (0.00)	3.00 (1.00)			
Day 21	Mean ± SD	4.76 ± 0.62	4.52 ± 0.75	0.251 ns	0.206 (0.010:0.480)	Small
	Median (IQR)	5.00 (0.00)	5.00 (1.00)			

Abbreviations: NA, not applicable; ns, not significant.

**Table 8:** Descriptive statistics for color match score.

Group	Time	Mean	SD	Median	Minimum	Maximum
Ozone	Baseline	0.00	0.00	0.00	0.00	0.00
	Day 3	2.90	0.83	3.00	1.00	4.00
	Day 7	4.52	1.03	5.00	2.00	6.00
	Day 14	6.62	1.20	7.00	4.00	9.00
	Day 21	8.52	1.03	9.00	7.00	10.00
	Day 42	9.95	0.22	10.00	9.00	10.00

HA	Baseline	0.00	0.00	0.00	0.00	0.00
	Day 3	3.05	0.86	3.00	2.00	4.00
	Day 7	4.81	1.21	5.00	2.00	7.00
	Day 14	6.14	1.39	6.00	3.00	9.00
	Day 21	7.76	1.30	8.00	5.00	10.00
	Day 42	10.00	0.00	10.00	10.00	10.00

**Table 9:** Intergroup comparisons and summary statistics for color match score.

Time	Measurement	Color match score		*p*‐Value	Effect size	
		Ozone	HA		Eta2 (h) (95% CI)	Magnitude
Baseline	Mean ± SD	0.00 ± 0.00	0.00 ± 0.00	NA	NA	NA
	Median (IQR)	0.00 (0.00)	0.00 (0.00)			
Day 3	Mean ± SD	2.90 ± 0.83	3.05 ± 0.86	0.641 ns	0.074 (0.006:0.390)	Small
	Median (IQR)	3.00 (1.00)	3.00 (2.00)			
Day 7	Mean ± SD	4.52 ± 1.03	4.81 ± 1.21	0.426 ns	0.158 (0.006:0.450)	Small
	Median (IQR)	5.00 (1.00)	5.00 (1.00)			
Day 14	Mean ± SD	6.62 ± 1.20	6.14 ± 1.39	0.426 ns	0.180 (0.008:0.460)	Small
	Median (IQR)	7.00 (1.00)	6.00 (2.00)			
Day 21	Mean ± SD	8.52 ± 1.03	7.76 ± 1.30	0.350 ns	0.282 (0.030:0.550)	Small
	Median (IQR)	9.00 (1.00)	8.00 (2.00)			
Day 42	Mean ± SD	9.95 ± 0.22	10.00 ± 0.00	0.426 ns	0.154 (0.120:0.320)	Small
	Median (IQR)	10.00 (0.00)	10.00 (0.00)			

Abbreviations: NA, not applicable; ns, not significant.

No adverse events, allergic reactions, or complications were observed in either treatment group during the study period. All participants completed the full 42‐day follow‐up without the need for additional medical intervention, unplanned treatment discontinuation, or escalation of analgesic use beyond the prescribed regimen.

## 4. Discussion

Patient reported outcomes are now of prime importance in periodontal plastic surgeries [16]. Several clinical trials have been conducted to minimize postoperative morbidity in patients undergoing soft tissue graft harvesting. Many of these studies yielded favorable results by utilizing various materials, including HA gel [6, 8], ozone therapy [10, 11] topical simvastatin gel [20], and advanced platelet‐rich fibrin (A‐PRF) [21]. These interventions have shown promising potential in enhancing postoperative recovery by reducing pain, promoting wound healing, and improving the overall esthetic integration of grafted tissues [1].

HA is widely favored for covering palatal wounds due to its unique anti‐inflammatory, antioxidant, and bacteriostatic properties. HA possesses roles in wound healing, reducing scarring, and supporting tissue regeneration by retaining moisture. Extensively studied in clinical trials [6, 8, 9], HA’s versatility in gel or spray form makes it a noninvasive, user‐friendly option. Thus, it is an effective dressing for managing palatal wounds after FGG harvesting.

Despite the growing interest in these treatment modalities, there remains a lack of data in the literature concerning the effect of ozone gel on the clinical parameters associated with patients with mucogingival defects following FGG harvesting. To the best of the authors’ knowledge, this study represents the first (RCT) evaluating the clinical effect of ozone gel application on palatal wounds after FGG harvesting. Additionally, it is the first study to compare ozone therapy with HA treatment with a large sample size, providing valuable insights into the potential advantages of these interventions.

In addition to their observed clinical effectiveness, the present study demonstrated that both ozone and HA gels were well tolerated, with no adverse events or complications reported among the enrolled participants. This outcome supports the excellent biocompatibility and local safety profile of both agents in intraoral wound healing, which is consistent with previously published clinical trials [8, 22]. Furthermore, the absence of complications may be attributed, in part, to the rigorous exclusion of medically compromised individuals and those with known hypersensitivities, as well as to standardized wound care protocols implemented during follow‐up. Direct clinical evaluation at all scheduled visits ensured systematic monitoring and allowed for confident reporting of the absence of treatment‐related morbidity.

Pain management is a critical aspect of postsurgical recovery, as it significantly influences patient comfort and compliance with postoperative care [16]. The data herein indicate a significant reduction in pain over time in both the ozone and HA groups. At baseline, both groups exhibited similar VAS scores (mean: 6.90), suggesting comparable initial pain levels. However, a trend of faster pain reduction was observed in the ozone group, with VAS scores dropping more rapidly by Day 3 (mean: 4.48) compared to the HA group (mean: 5.24). Although this difference was not statistically significant (*p* > 0.05), it implies a potential advantage of ozone therapy in early pain alleviation. By Day 14, both groups had reached near‐zero pain levels, confirming the effectiveness of both interventions in long‐term pain control. The moderate effect size observed at Day 7 further supports the clinical relevance of the faster pain reduction in the ozone group.

These findings align with previous studies [10, 11, 22, 23] suggesting that ozone therapy has analgesic properties, potentially due to its antimicrobial effects and ability to stimulate tissue oxygenation. The acceleration of pain relief with ozone therapy may be attributed to its ability to modulate inflammatory responses, thereby reducing nociceptive signaling at the wound site.

The observed early postoperative pain relief in the ozone‐treated group, particularly notable by Day 3, aligns with previous RCTs evaluating ozone’s analgesic efficacy in periodontal surgeries [10, 11]. Such studies have similarly reported a significant reduction in early postoperative pain scores attributed to ozone’s anti‐inflammatory and antimicrobial properties, which likely attenuate the inflammatory mediators responsible for acute pain responses [10, 11, 24]. Conversely, HA, despite its established benefits in mucosal wound healing, may provide slower analgesic effects given its primary action through enhancing epithelialization and modulating inflammation over an extended period [6, 8]. This comparative analysis provides valuable clinical context, reinforcing ozone’s potential advantages for immediate postoperative comfort.

Wound healing is a multifaceted process involving cellular proliferation, tissue remodeling, and angiogenesis. The healing index demonstrated a consistent improvement over time for both treatment groups. Initially, both groups had a baseline score of 1.00, reflecting the absence of healing. By Day 3, the ozone group exhibited a greater increase in the healing index (mean: 2.10) compared to the HA group (mean: 1.62). This pattern persisted, with the ozone group maintaining a slightly higher healing index than the HA group throughout the study period. By Day 21, the healing index for the ozone group was 4.76, whereas the HA group reached 4.52. Although the intergroup differences were not statistically significant, the moderate effect size observed at specific time points suggests a potential advantage of ozone therapy in accelerating the healing process.

The intragroup analysis confirmed that both treatments significantly improved healing over time (*p* < 0.001), with large effect sizes, indicating that both interventions were effective in promoting tissue recovery. These findings reinforce the hypothesis that ozone therapy, due to its antimicrobial and proangiogenic properties, may contribute to faster re‐epithelialization and tissue repair, providing a potential advantage in clinical practice [11, 23].

Regarding wound healing dynamics, both treatment modalities in our study demonstrated steady improvement over the follow‐up period. However, slightly superior, though statistically nonsignificant, Landry healing index scores observed in the ozone‐treated group on Days 3 and 14 correspond with prior clinical findings indicating accelerated mucosal repair with ozone application [10, 22]. Ozone’s mechanisms, including stimulation of local angiogenesis, enhanced tissue oxygenation, and reduced microbial load, provide plausible explanations for improved early healing outcomes [24]. Similarly, HA gel facilitated progressive healing, supporting earlier literature which emphasizes its role in cellular proliferation, migration, and inflammation reduction [6, 8]. These comparative insights underscore both agents’ validity, with subtle distinctions possibly relevant in clinical decision‐making scenarios.

Esthetic outcomes, as assessed by the color match score, comprised an important aspect of the current study. Patients undergoing soft tissue grafting often prioritize the final appearance of the treated area, making tissue color matching a crucial parameter. Both groups demonstrated a gradual increase in tissue color matching over time, with no significant differences between them. By Day 42, both the ozone and HA groups had achieved a near‐perfect color match (median: 10.00), suggesting that both treatments facilitate excellent esthetic recovery.

However, a slight advantage was noted in the ozone group on Days 14 and 21, where the median color match scores were higher than those in the HA group. This difference, though not statistically significant, may indicate a more rapid tissue adaptation and integration with ozone therapy. The strong positive correlation (*r*s = 0.897, *p* < 0.001) between the healing index and color match score further reinforces the idea that improved healing is associated with better tissue esthetics. This supports the consideration of ozone therapy as a viable option for patients who seek faster and more visually appealing recovery.

The role of topical biologic agents, such as ozone and HA, is increasingly acknowledged in contemporary mucogingival surgical protocols, given their potential to enhance postoperative recovery and patient comfort. Previous comparative studies employing other biologic agents like platelet‐rich fibrin (PRF) and simvastatin gels have demonstrated significant benefits in pain relief, wound healing, and patient‐reported esthetic satisfaction [20, 21]. In this context, our findings contribute novel comparative data suggesting ozone gel’s favorable profile as a biologically active adjunct with specific early‐phase advantages. However, the lack of significant statistical differentiation in key outcomes indicates the necessity for larger‐scale studies directly comparing ozone with established biologics, to clearly define its relative efficacy and optimal clinical indications.

Analgesic intake serves as a secondary indicator of postoperative pain levels and patient discomfort. Analgesic consumption was significantly lower in the ozone group compared to the HA group, particularly on Day 2 (*p* = 0.042) and Day 3 (*p* = 0.042). This finding aligns with the observed pain reduction trend, where the ozone group experienced a more rapid decline in VAS scores. The decreased need for analgesics suggests that ozone therapy may provide superior analgesic effects, reducing patient dependency on medication. From Day 4 onward, analgesic intake was negligible in both groups, indicating that both interventions effectively controlled postoperative pain by the mid‐recovery phase.

The moderate effect size onDays 2 and 3 highlights the potential clinical significance of this finding, suggesting that ozone therapy may be particularly beneficial in the immediate postoperative period. These results further emphasize the potential for ozone therapy to serve as an effective nonpharmacological alternative for pain management, minimizing reliance on conventional analgesics, which often carry risks of gastrointestinal side effects and dependency.

Although differences between ozone and HA groups were not statistically significant, moderate effect sizes observed on Day 7 (pain scores) and Days 2 and 3 (analgesic consumption) indicate potential early postoperative advantages of ozone therapy that may not be fully represented by *p*‐values alone [25]. Given sufficient statistical power, these findings reflect clinically meaningful distinctions between two effective interventions. Ozone therapy’s established biological properties, such as antimicrobial, anti‐inflammatory, and tissue oxygenation effects [10, 26], likely contribute to its potential for enhanced early pain relief and reduced analgesic use, supporting its adjunctive role in palatal wound management.

The findings of this study have significant clinical implications for postoperative management strategies. The comparable efficacy of ozone therapy and HA in pain reduction, healing, and esthetic recovery suggests that both treatments are viable options. However, the slight advantages observed in the ozone group, particularly in early pain relief, reduced analgesic dependence, and accelerated healing, suggest that ozone therapy may be a preferable choice for clinicians seeking rapid postoperative recovery with minimal pharmacological intervention.

The results of this study demonstrated a significant reduction in pain levels over time in both treatment groups. While both groups exhibited similar initial VAS scores, a faster decrease in pain was observed in the ozone group by Day 3. This trend is consistent with previous studies [11, 24] that have reported the analgesic effects of ozone therapy. These studies suggested that ozone therapy promotes pain relief through its antimicrobial properties and its ability to enhance tissue oxygenation, which in turn modulates inflammatory responses and reduces nociceptive signaling at the wound site.

Although the difference in pain reduction between the ozone and HA groups was not statistically significant (*p* > 0.05), the observed trend suggests a potential advantage of ozone therapy in accelerating early postoperative pain relief. This supports previous trials [10, 11, 26] indicating that ozone may act as an adjunctive analgesic agent in periodontal surgical procedures. However, further research with a larger sample size may be required to determine whether these effects reach statistical significance.

In this study, both HA and ozone therapy demonstrated a positive impact on wound healing, with a steady improvement in the healing index over time. The ozone group exhibited a slightly higher healing index than the HA group at all time points, with the most notable differences observed on Day 3 and 7.

These findings agree with previous studies [10, 11] on the wound‐healing properties of ozone therapy which have emphasized its ability to accelerate re‐epithelialization and tissue repair through antimicrobial and proangiogenic mechanisms. Furthermore, the observed healing enhancement in the HA group is consistent with prior research [6, 8] that identified HA as an effective material for managing palatal wounds due to its anti‐inflammatory, antiedematous, and tissue‐regenerative properties. These studies demonstrated that HA, whether applied in gel or spray form, serves as a reliable noninvasive treatment option for wound management, further corroborating the results of the present study.

Despite the observed advantages of ozone therapy in wound healing, the intergroup differences did not reach statistical significance. This is in contrast with some previous studies [6, 8] on HA, which reported statistically significant improvements in healing parameters. The discrepancy may be attributed to differences in study design, sample size, or the severity of surgical wounds.

While HA has been extensively studied in periodontal wound healing [6, 8, 9, 21, 27], there remains a notable gap in the literature regarding the effects of ozone gel, specifically in the management of palatal wounds after FGG harvesting. Unlike previous studies that have investigated the impact of HA and other materials, such as topical simvastatin gel [20] and advanced platelet‐rich fibrin (A‐PRF) [21], there is a lack of data directly comparing ozone therapy with HA in this context. Therefore, this study provides valuable insights into the potential role of ozone as an alternative or adjunctive treatment for mucogingival wound healing.

Furthermore, while prior studies have highlighted the efficacy of ozone in reducing postoperative pain and inflammation [10, 22], their focus has predominantly been on other periodontal applications, such as nonsurgical periodontal therapy or implant site preparation [22]. Additionally, since this study is among the first randomized controlled clinical trials to evaluate the application of ozone gel on palatal wounds after FGG harvesting, further research is warranted to further explore and validate its clinical effects. By specifically evaluating ozone therapy for palatal wound healing, this study contributes novel data to the existing body of knowledge and lays the foundation for future research in this area.

The present study evaluated postoperative pain, analgesic consumption, wound healing indices, and color match outcomes; however, patient‐reported outcomes (PROs) were not assessed, limiting a comprehensive understanding of treatment effectiveness from the patient’s perspective, as emphasized by recent consensus statements [28, 29]. Validated PROs were not assessed, limiting patient‐centered evaluation. Formal reliability testing of photographic documentation was not conducted [30]. Additionally, the study primarily relied on subjective indices (Landry healing index, VAS), without employing objective methods such as the hydrogen peroxide bubbling test or digital planimetric analysis, due to logistical constraints [31].

Although dosage and application frequency were standardized, tissue penetration and dose–response were not quantitatively evaluated. The relatively small sample size, despite being based on power analysis, may have limited the detection of subtle differences. Future studies with larger samples should incorporate objective biochemical and physiological markers as interleukin‐1*β* (IL‐1*β*) and vascular endothelial growth factor (VEGF), including oxidative stress indicators, to ensure methodological rigor and comprehensive evaluation.

## 5. Recommendations for Future Research

Future studies with extended follow‐up periods are needed to assess the long‐term benefits of ozone therapy and HA application, particularly in relation to tissue stability and the recurrence of mucogingival defects. Investigating the underlying biological mechanisms responsible for the observed effects will help to better understand the molecular and cellular pathways involved in ozone‐induced healing. Incorporating PROs and quality‐of‐life assessments would provide a more comprehensive evaluation of these therapies in clinical practice. Moreover, future trials should also compare ozone therapy and HA with other biologics, such as growth factors and stem cell‐based approaches, to explore further improvements in wound healing strategies.

## 6. Conclusions

Both ozone therapy and HA were effective in managing postoperative pain, promoting wound healing, and enhancing tissue esthetics. The observed trends suggest a potential advantage of ozone therapy in early pain reduction and healing acceleration. These findings support the incorporation of ozone therapy as a promising alternative or adjunct to conventional treatments for postoperative recovery, warranting further investigation in larger clinical trials. By offering an effective and potentially superior approach to wound management, ozone therapy holds promise in optimizing patient outcomes following soft tissue grafting procedures.

With a growing emphasis on minimally invasive and biologically driven treatment strategies, the integration of HA and ozone therapy into clinical practice could revolutionize postsurgical healing following FGG procedures. By enhancing healing efficiency and improving patient comfort, these therapies hold significant promise for broader applications in periodontal and implant‐related soft tissue surgeries.

## Conflicts of Interest

The authors declare no conflicts of interest.

## Author Contributions

Hisham Tarek: conception and design of the study, patient recruitment, clinical procedures, data acquisition, and drafting of the manuscript. Dalia Ghalwash: supervision, methodological guidance, and critical revision of the manuscript for important intellectual content. Nesma Shemais: clinical guidance, data analysis, and manuscript review and editing. Ahmed El Barbary: statistical analysis, interpretation of data, and critical revision of the manuscript.

## Funding

This research received no external funding and was entirely self‐funded by the authors. The study was conducted as part of our independent academic work, with no financial support from any organization or institution.

## Data Availability

The data that support the findings of this study are available from the corresponding author upon reasonable request.

## References

[1] Tavelli L. , Barootchi S. , Stefanini M. , Zucchelli G. , Giannobile W. V. , and Wang H. L. , Wound Healing Dynamics, Morbidity, and Complications of Palatal Soft-Tissue Harvesting, Periodontology 2000. (2023) 92, no. 1, 90–119, 10.1111/prd.12466. 36583690

[2] Sullivan H. C. and Atkins J. H. , Free Autogenous Gingival Grafts. I. Principles of Successful Grafting, Periodontics. (1968) 6, no. 3, 121–129. 5240496

[3] Wyrębek B. , Górski B. , and Górska R. , Patient Morbidity at the Palatal Donor Site Depending on Gingival Graft Dimension, Dental and Medical Problems. (2018) 55, no. 2, 153–159, 10.17219/dmp/91406, 2-s2.0-85049217307. 30152618

[4] Tavelli L. , Ravidà A. , and Saleh M. H. , et al.Pain Perception Following Epithelialized Gingival Graft Harvesting: A Randomized Clinical Trial, Clinical Oral Investigations. (2019) 23, no. 1, 459–468, 10.1007/s00784-018-2455-5, 2-s2.0-85056260731. 29713890

[5] Lektemur Alpan A. and Torumtay Cin G. , PRF Improves Wound Healing and Postoperative Discomfort After Harvesting Subepithelial Connective Tissue Graft From Palate: A Randomized Controlled Trial, Clinical Oral Investigations. (2020) 24, no. 1, 425–436, 10.1007/s00784-019-02934-9, 2-s2.0-85066107817. 31104113

[6] Yıldırım S. , Özener H.Ö. , Doğan B. , and Kuru B. , Effect of Topically Applied Hyaluronic Acid on Pain and Palatal Epithelial Wound Healing: An Examiner-Masked, Randomized, Controlled Clinical Trial, Journal of Periodontology. (2018) 89, no. 1, 36–45, 10.1902/jop.2017.170105, 2-s2.0-85041730848. 28914592

[7] Ugazio E. , Tullio V. , Binello A. , Tagliapietra S. , and Dosio F. , Ozonated Oils as Antimicrobial Systems in Topical Applications. Their Characterization, Current Applications, and Advances in Improved Delivery Techniques, Molecules. (2020) 25, no. 2, 10.3390/molecules25020334, 334. 31947580 PMC7024311

[8] Hassan A. , Ahmed E. , Ghalwash D. , and Elarab A. E. , Clinical Comparison of MEBO and Hyaluronic Acid Gel in the Management of Pain After Free Gingival Graft Harvesting: A Randomized Clinical Trial, International Journal of Dentistry. (2021) 2021, no. 1, 10.1155/2021/2548665, 2548665. 34426739 PMC8380183

[9] Asparuhova M. B. , Kiryak D. , Eliezer M. , Mihov D. , and Sculean A. , Activity of Two Hyaluronan Preparations on Primary Human Oral Fibroblasts, Journal of Periodontal Research. (2019) 54, no. 1, 33–45, 10.1111/jre.12602, 2-s2.0-85053920943. 30264516 PMC6586051

[10] Taşdemir Z. , Alkan B. A. , and Albayrak H. , Effects of Ozone Therapy on the Early Healing Period of Deepithelialized Gingival Grafts: A Randomized Placebo-Controlled Clinical Trial, Journal of Periodontology. (2016) 87, no. 6, 663–671, 10.1902/jop.2016.150217, 2-s2.0-84971219384. 26777769

[11] Al-Sherbini O. M. , Elbattawy W. A. , and Hosny M. M. , The Effect of Ozone Therapy on Pain Perception After Free Gingival Graft Surgery in Patients With Mucogingival Defects. A Randomized Controlled Clinical Trial, Advanced Dental Journal. (2024) 6, no. 4, 789–799, 10.21608/adjc.2024.248630.1426.

[12] Schulz K. F. , CONSORT 2010 Statement: Updated Guidelines for Reporting Parallel Group Randomized Trials, Annals of Internal Medicine. (2010) 152, 1–7. 20335313 10.7326/0003-4819-152-11-201006010-00232

[13] Camargo P. M. , Melnick P. R. , and Kenney E. B. , The Use of Free Gingival Grafts for Aesthetic Purposes, Periodontology 2000. (2001) 27, no. 1, 72–96, 10.1034/j.1600-0757.2001.027001072.x, 2-s2.0-0345859674. 11551301

[14] Zucchelli G. , Tavelli L. , and McGuire M. K. , et al.Autogenous Soft Tissue Grafting for Periodontal and Peri-Implant Plastic Surgical Reconstruction, Journal of Periodontology. (2020) 91, no. 1, 9–16, 10.1002/JPER.19-0350. 31461778

[15] Price D. D. , McGrath P. A. , Rafii A. , and Buckingham B. , The Validation of Visual Analogue Scales as Ratio Scale Measures for Chronic and Experimental Pain, Pain. (1983) 17, no. 1, 45–56, 10.1016/0304-3959(83)90126-4, 2-s2.0-0020565258. 6226917

[16] Zucchelli G. , Mele M. , and Stefanini M. , et al.Patient Morbidity and Root Coverage Outcome After Subepithelial Connective Tissue and De-Epithelialized Grafts: A Comparative Randomized-Controlled Clinical Trial, Journal of Clinical Periodontology. (2010) 37, no. 8, 728–738, 10.1111/j.1600-051X.2010.01550.x, 2-s2.0-77954592714. 20590963

[17] Landry R. G. , Effectiveness of Benzydamine HC1 in the Treatment of Periodontal Post-Surgical Patients (Doctoral Dissertation), 1985.

[18] Nandhra K. , Woolley J. , Bister D. , Sherriff M. , and Jeremiah H. , Smartphones or Digital SLRs for Clinical Dental Photography: Is There a Difference?, European Journal of Orthodontics. (2025) 47, no. 4, 10.1093/ejo/cjaf033, cjaf033. 40501276

[19] Fernández-Coll A. P. , Garcés-Elías M. C. , Beltrán J. A. , León-Manco R. A. , and Mas-López J. , Validity and Reliability According to the Type of Examiners in the Process of Calibrating Dental Caries Experience Using the DMFT Index, Methods and Protocols. (2024) 7, no. 5, 10.3390/mps7050083, 83. 39452797 PMC11510506

[20] Madi M. and Kassem A. , Topical Simvastatin Gel as a Novel Therapeutic Modality for Palatal Donor Site Wound Healing Following Free Gingival Graft Procedure, Acta Odontologica Scandinavica. (2018) 76, no. 3, 212–219, 10.1080/00016357.2017.1403648, 2-s2.0-85034266098. 29145771

[21] Sousa F. , Machado V. , Botelho J. , Proença L. , Mendes J. J. , and Alves R. , Effect of A-PRF Application on Palatal Wound Healing After Free Gingival Graft Harvesting: A Prospective Randomized Study, European Journal of Dentistry. (2020) 14, no. 1, 063–069, 10.1055/s-0040-1702259. PMC7069756 32168533

[22] Gupta G. and Mansi B. , Ozone Therapy in Periodontics, Journal of Medicine and Life. (2012) 5, no. 1, 59–67. 22574088 PMC3307081

[23] McGuire M. K. , Scheyer E. T. , and Gwaltney C. , Commentary: Incorporating Patient-Reported Outcomes in Periodontal Clinical Trials, Journal of Periodontology. (2014) 85, no. 10, 1313–1319, 10.1902/jop.2014.130693, 2-s2.0-84907494842. 25034790

[24] El Meligy O. A. , Elemam N. M. , and Talaat I. M. , Ozone Therapy in Medicine and Dentistry: A Review of the Literature, Dentistry Journal. (2023) 11, no. 8, 10.3390/dj11080187, 187. 37623283 PMC10453584

[25] Sullivan G. M. and Feinn R. , Using Effect Size—or Why the P Value is Not Enough, Journal of Graduate Medical Education. (2012) 4, no. 3, 279–282, 10.4300/JGME-D-12-00156.1. 23997866 PMC3444174

[26] Barahim A. A. , Shemais N. , Mousa A. , and Darhous M. , Clinical and Radiographic Evaluation of Non-Surgical Therapy With and Without Ozone Gel Application in Controlled Type 2 Diabetic Patients With Periodontitis: A Randomized Controlled Clinical Trial, BMC Oral Health. (2024) 24, no. 1, 10.1186/s12903-024-05212-7, 1435. 39587593 PMC11590345

[27] Fawzy El-Sayed K. M. , Dahaba M. A. , Aboul-Ela S. , and Darhous M. S. , Local Application of Hyaluronan Gel in Conjunction With Periodontal Surgery: A Randomized Controlled Trial, Clinical Oral Investigations. (2012) 16, no. 4, 1229–1236, 10.1007/s00784-011-0630-z, 2-s2.0-84864282928. 22012469

[28] Wittneben J. G. , Wismeijer D. , Brägger U. , Joda T. , and Abou-Ayash S. , Patient-Reported Outcome Measures Focusing on Aesthetics of Implant-and Tooth-supported Fixed Dental Prostheses: A Systematic Review and Meta-analysis, Clinical Oral Implants Research. (2018) 29, 224–240. 10.1111/clr.13295 30328183

[29] Rener-Sitar K. , John M. T. , Truong V. , Tambe S. , and Theis-Mahon N. , Nonmalignant Oral Disease–Specific Dental Patient-Reported Outcome Measures for Adult Patients: A Systematic Review, Journal of Evidence Based Dental Practice. (2021) 21, no. 1, 101529. 34051957 10.1016/j.jebdp.2021.101529

[30] Signori C. , Collares K. , Cumerlato C. B. , Correa M. B. , Opdam N. J. , and Cenci M. S. , Validation of Assessment of Intraoral Digital Photography for Evaluation of Dental Restorations in Clinical Research, Journal of Dentistry. (2018) 71, 54–60. 29438796 10.1016/j.jdent.2018.02.001

[31] Kızıltoprak M. and Uslu M.Ö. , Comparison of the Effects of Injectable Platelet-Rich Fibrin and Autologous Fibrin Glue Applications on Palatal Wound Healing: A Randomized Controlled Clinical Trial, Clinical Oral Investigations. (2020) 24, 4549–4561. 32424462 10.1007/s00784-020-03320-6

